# Prognostic and Therapeutic Utility of Variably Expressed Cell Surface Receptors in Osteosarcoma

**DOI:** 10.1155/2021/8324348

**Published:** 2021-02-02

**Authors:** Yoav Zvi, Elif Ugur, Brian Batko, Jonathan Gill, Michael Roth, Richard Gorlick, David Hall, Janet Tingling, Donald A. Barkauskas, Jinghang Zhang, Rui Yang, Bang H. Hoang, David S. Geller

**Affiliations:** ^1^Department of Orthopaedic Surgery, Montefiore Medical Center, The Children's Hospital at Montefiore, Bronx, NY, USA; ^2^The Albert Einstein College of Medicine, Bronx, NY, USA; ^3^Department of Pediatrics, MD Anderson Cancer Center, Houston, TX, USA; ^4^QuadW-COG Childhood Sarcoma Biostatistics and Annotation Office, Children's Oncology Group, Monrovia, CA, USA; ^5^Department of Preventive Medicine, Keck School of Medicine of the University of Southern California, Los Angeles, CA, USA; ^6^Flow Cytometry Core, Albert Einstein College of Medicine, Bronx, NY, USA

## Abstract

**Background:**

Six cell surface receptors, human epidermal growth factor receptor-2 (Her-2), platelet-derived growth factor receptor-*β* (PDGFR-*β*), insulin-like growth factor-1 receptor (IGF-1R), insulin receptor (IR), c-Met, and vascular endothelial growth factor receptor-3 (VEGFR-3), previously demonstrated variable expression across varying patient-derived and standard osteosarcoma (OS) cell lines. The current study sought to validate previous expression patterns and evaluate whether these receptors offer prognostic and/or therapeutic value.

**Methods:**

Patient-derived OS cell lines (*n* = 52) were labeled with antibodies to Her-2, PDGFR-*β*, IGF-1R, IR, c-Met, and VEGFR-3. Expression was characterized using flow cytometry. The difference in geometric mean fluorescent intensity (geoMFI_diff_ = geoMFI_positive_ − geoMFI_negative_) was calculated for each receptor across all cell lines. Receptor expression was categorized as low (Q1), intermediate (Q2, Q3), or high (Q4). The event-free survival (EFS) and overall survival for the six cell surface receptors were estimated by the Kaplan–Meier method. Differences in hazard for EFS event and overall survival event for patients in each of the three expression levels in each of the six cell surface receptors were assessed using the log-rank test.

**Results:**

All 6 receptors were variably expressed in the majority of cell lines. IR and PDGFR-*β* expressions were found to be significant predictors for EFS amongst patients with nonmetastatic disease (*p*=0.02 and 0.01, respectively). The hazard ratio for EFS was significantly higher between high IR and intermediate IR expression (HR = 2.66, *p*=0.02), as well as between high PDGFR-*β* and intermediate PDGFR-*β* expression (HR = 5.68, *p*=0.002). Her-2, c-Met, IGF-1R, and VEGFR-3 were not found to be significant predictors for either EFS or overall survival.

**Conclusion:**

The six cell surface receptors demonstrated variable expression across the majority of patient-derived OS cell lines tested. Limited prognostic value was offered by IR and PDGFR-*β* expression within nonmetastatic patients. The remaining receptors do not provide clear prognostic utility. Nevertheless, their consistent, albeit variable, surface expression across a large panel of patient-derived OS cell lines maintains their potential use as future therapeutic targets.

## 1. Introduction

Osteosarcoma (OS) is the most common nonhematologic primary bone malignancy and the fifth most common primary malignancy among adolescents and young adults [1, 2]. The overall 5-year survival has plateaued at roughly 70% and has not improved in nearly four decades [2–5]. Multiple cooperative efforts including the recent EURAMOS-1 trial as well as studies by the European Osteosarcoma Intergroup [6, 7] have repeatedly demonstrated that intensifying conventional chemotherapy alone is futile, underscoring the need for novel approaches. Toward that end, there remains an ongoing interest in identifying OS biomarkers that can be leveraged for either prognostication and/or as therapeutic targets.

It is valuable to distinguish inhibiting receptor and its associated pathway from using a receptor as a means of targeting the expressing cell. The former approach needs the pathway to be functional and critical if the therapeutic measure is to have an impact. The latter approach is pathway-independent and uses the receptor solely for directing the therapeutic agent to the cell of interest. Targeting can be accomplished using a variety of means, including radioimmunotherapy and antibody-drug conjugates, both of which have been of interest in the setting of OS. While uniquely expressed receptors or receptor patterns are ideal, consistent expression and overexpression may offer targeting opportunities, independent of associated intracellular pathways. A comprehensive understanding of osteosarcoma's surfaceome may prove increasingly useful and is ongoing [8].

The cell surface receptor expression pattern in OS was previously studied using patient-derived and standard OS cell lines [9]. Insulin-like growth factor receptor 2 (IGF2R) was consistently overexpressed across all cell lines evaluated and further investigated as a potential novel therapeutic target using radioimmunotherapy [10, 11]. A second group of receptors including human epidermal growth factor receptor-2 (HER-2), platelet-derived growth factor receptor-*β* (PDGFR-*β*), IGF-1R, insulin receptor (IR), c-Met, and vascular endothelial growth factor receptor (VEGFR)-3 was found to be variably expressed. Consideration was given as to whether expression across these receptors could yield either prognostic and/or therapeutic utility for subsets of OS tumors.

The primary purpose of this study was to address the following questions: (1) Does the variable pattern of receptor expression corroborate previously reported findings? (2) Does the level of surface receptor expression provide prognostic utility?

## 2. Materials and Methods

### 2.1. Cell Lines

Ninety-nine OS patient-derived cell lines were obtained from the Children's Oncology Group (COG) Biorepository (AOST16B4-Q). The Cooperative Human Tissue Network (CHTN), which performs the banking function for COG, prepared H&D sections confirming the presence of osteosarcoma tissue. All cell lines originated from patients with high-grade localized OS and were banked following informed written patient consent and Institutional Review Board (IRB) approval. Clinical outcomes for each patient were blinded to the investigators and only associated at the time of analysis. Of these 99 patients, 50 patients survived and 49 patients had died. Eighteen cell lines did not grow in culture, leaving 81 cell lines available for analysis. Fifty-two cell lines yielded a sufficient number of cells for data analysis. Forty-seven patients had survival data. One patient had 2 observations that were treated as independent observations. The survival and hazard ratio analyses were performed using these 48 observations.

### 2.2. Flow Cytometry Analysis

Cells were thawed and centrifuged to remove their freezing medium. After resuspension with MEM-*α* media and 10% fetal bovine serum, cells were counted to determine the number of live cells per sample. Approximately 1 million live cells were stained and assessed using flow cytometry.

Cell staining was done using commercially available antihuman antibodies to Her-2, IGF-1R, IR, VEGFR-3, c-MET, and PDGFR-*β* receptors in accordance with manufacture instructions (Table 1). Each antibody was conjugated to one of three fluorophores: phycoerythrin (PE), fluorescein-isothiocyanate (FITC), or allophycocyanin (APC) (Table 1). Control tubes were also prepared using isotype-matched antibodies for each sample.

Flow cytometry analysis was performed using a Becton Dickinson LSRII digital benchtop flow cytometer (Becton Dickinson, Mountain View, CA). To gate for single live cells, standard forward and side scatter gating protocols with DAPI staining were employed as follows: FSC-A/SSC-A, FSC-A/FSC-H, SSC-A/SSC-H, and SSC-A/DAPI. A minimum of 1,000 single live cells was required for analysis. Data were analyzed using FlowJo software (BD Biosciences, Franklin Lakes, NJ).

### 2.3. Receptor Characterization

Receptor expression was plotted across all assayed cell lines and expressed relative to a negative control. The negative controls used for all six cell surface receptors were their respective isotype-matched controls. For each cell line, analyses were conducted in triplicate and averaged, to mitigate the impact of unrealized technical error. The geometric mean fluorescent intensity (geoMFI) was calculated for each receptor and for each isotype-matched negative control across all cell lines.

The primary parameter for all surface receptors was the difference in geoMFI from positive and negative controls: geoMFI_diff_ = geoMFI_positive_ − geoMFI_negative_. Each receptor across all cell lines was then categorized into a low, intermediate, or high expression group. The low expression group included all receptors whose geoMFI_diff_ fell within the first quartile, the intermediate expression group included all receptors whose geoMFI_diff_ fell within the second and third quartiles, and the high expression group included all receptors whose geoMFI_diff_ fell within the fourth quartile.

### 2.4. Statistical Analysis

Each pair of characteristics was checked for association within the analytic population by the exact conditional test of proportions. Age at enrollment was checked in this manner as a categorical variable (0–9, ≥10 years) and also as a continuous variable using the *t*-test or a one-way analysis of variance (ANOVA), as appropriate.

The outcome in terms of EFS and overall survival was compared among the groups defined by the demographic variables. Event-free survival was defined as days from enrollment either to an event (relapse/progression, SMN, or death) or to the last contact. For EFS, patients were considered censored at last contact if they did not experience an event. Overall survival was defined as days from enrollment until either death or last contact. Osteosarcoma patients were considered censored at last contact if they were alive at that time.

The EFS and overall survival for the 6 receptors were estimated by the Kaplan–Meier method and stratified by metastatic status at the time of diagnosis. Differences in hazard for EFS event and overall survival event across all cell lines for the 6 cell surface receptors were assessed using the log-rank test. Analyses were done in SAS9.4 using PROC LIFETEST and PROC FREQ.

## 3. Results

Of the 48 patients included in the final analysis, 39 (81.3%) were above the age of 10 years old. Metastatic disease at initial diagnosis was present in 17 (35.4%) patients. A summary of captured demographic patient data is provided in Table 2. Metastatic status at diagnosis was found to be significantly associated with overall survival (*p*=0.01), but not with EFS. None of the other demographic variables were found to be significant for either EFS or overall survival. Supplementary Tables 1 and 2 summarize the number of EFS and overall survival events with respect to low, intermediate, or high receptor expression.

**Figure 1:**
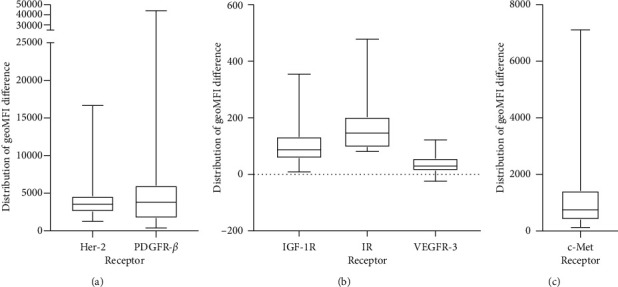
Distribution of expression across all cell lines for Her-2 and PDGFR-*β* (a), IGF-1R, IR, and VEGFR-3 (b), and c-Met (c), expressed as geoMFI_diff_.

The majority of cell lines were found to express the receptors of interest when compared to their negative control. The distribution of geoMFI_diff_ for each surface receptor demonstrated substantial variability across all cell lines, with 3 receptors demonstrating wide variability (Her-2, PDGFR-*β*, and c-Met), 2 demonstrating moderate variability (IGF-1R and IR), and 1 demonstrating modest variability (VEGFR-3) (Figure 1 and Supplementary Figure 1). The raw geoMFI_diff_ between surface receptors and their respective negative controls are summarized in Supplementary Table 3. Expression data for each cell line, which were categorized as being low, intermediate, or high expression, are summarized in Supplementary Table 4. Of note, cell lines C204, C243, and C340 yielded negative geoMFI_diff_ values, a function of very low surface receptor expression relative to the cell lines' innate autofluorescence.

**Figure 2:**
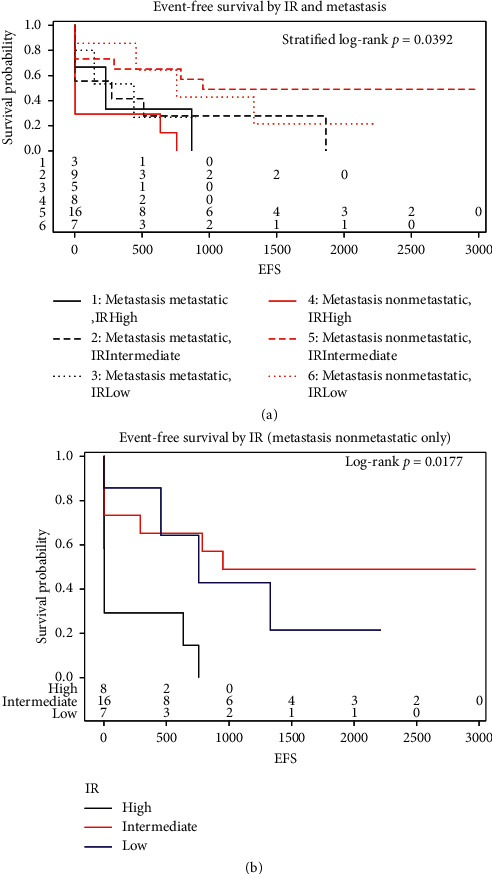
IR level was found to be a significant predictor for EFS when stratified by metastatic status at diagnosis; *p*=0.0392 (a). IR was found to be a significant predictor for EFS amongst patients with nonmetastatic disease at diagnosis; *p*=0.0177 (b).

IR level was found to be a significant predictor for EFS when stratified by metastatic status at diagnosis (*p* value = 0.04). Further analysis found IR level to be a significant predictor for EFS amongst patients whose disease was nonmetastatic at diagnosis (*p* value = 0.02) but was not found to be a significant predictor amongst patients whose disease was metastatic at diagnosis (*p* value = 0.77). The EFS hazard ratio was found to be significant between the high and intermediate levels of IR for all patients (HR high/intermediate = 2.656, *p* value = 0.02) and for nonmetastatic patients only (HR high/intermediate = 4.477, *p* value = 0.01). All other hazard ratios were not found to be significant (Figure 2 and Supplementary Table 5).

IR level was not found to be a significant predictor for overall survival when stratified by metastatic status at diagnosis (*p* value = 0.32). Further analysis did not find IR level to be a significant predictor for overall survival amongst patients whose disease was nonmetastatic at diagnosis (*p* value = 0.48), nor amongst patients whose disease was metastatic at diagnosis (*p* value = 0.59). None of the overall survival hazard ratios between levels of IR were found to be significant (Supplementary Table 6).

**Figure 3:**
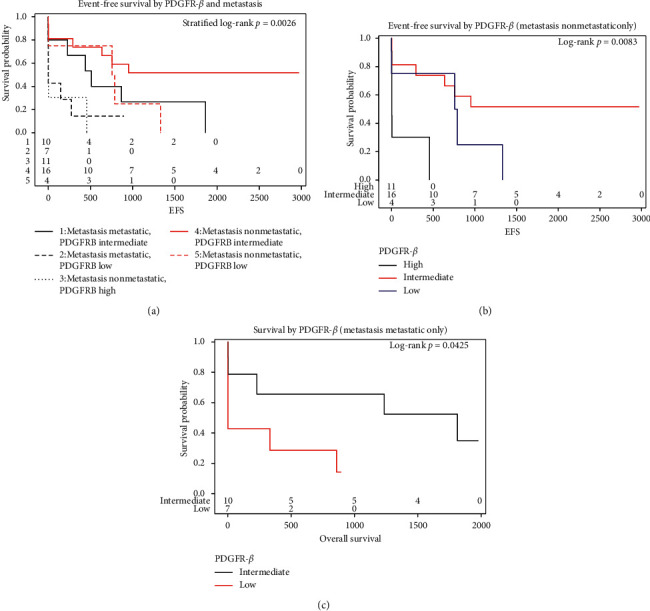
PDGFR-*β* level was found to be a significant predictor for EFS when stratified by metastatic status at diagnosis; *p*=0.0026 (a). PDGFR-*β* level was found to be a significant predictor for EFS amongst patients with nonmetastatic disease at diagnosis; *p*=0.0083 (b). PDGFR-*β* level was found to be a significant predictor for OS amongst patients with metastatic disease at diagnosis; *p*=0.0425 (c).

PDGFR-*β* level was found to be a significant predictor for EFS when stratified by metastatic status at diagnosis (*p* value = 0.003). Further analysis found PDGFR-*β* level to be a significant predictor for EFS amongst patients whose disease was nonmetastatic at diagnosis (*p* value = 0.01), but PDGFR-*β* level was not found to be a significant predictor amongst patients whose disease was metastatic at diagnosis (*p* value = 0.13). The EFS hazard ratios were found to be significant between the intermediate and low levels of PDGFR-*β* (HR intermediate/low = 0.377, *p* value = 0.02) and the high and intermediate levels of PDGFR-*β* (HR high/intermediate = 5.678, *p* value = 0.003) amongst all patients. The EFS hazard ratio was found to be significant between the high and intermediate levels of PDGFR-*β* (HR high/intermediate = 6.254, *p* value = 0.005) for nonmetastatic patients only. All other hazard ratios were not found to be significant (Figure 3 and Supplementary Table 7).

Further analysis found PDGFR-*β* level to be a significant predictor for overall survival amongst patients whose disease was metastatic at diagnosis (*p* value = 0.04), but PDGFR-*β* level was not found to be a significant predictor for overall survival amongst patients whose disease was nonmetastatic at diagnosis (*p* value = 0.55). The overall survival hazard ratios were found to be significant between the intermediate and low levels of PDGFR-*β* for all patients (HR intermediate/low = 0.310, *p* value = 0.02). All other hazard ratios were not found to be significant (Supplementary Table 8).

Expression levels for the remaining four receptors, Her-2, IGF-1R, c-Met, and VEGFR-3, were not found to be significant predictors for either EFS or overall survival when stratifying for metastatic status at diagnosis.

## 4. Discussion

In this study, Her-2, PDGFR-*β*, IGF-1R, IR, c-Met, and VEGFR-3 were variably expressed, reaffirming previously reported results [9]. Admittedly, methodology differed slightly in that findings in the current study were benchmarked against a negative isotype control rather than against a positive control. However, whereas the previous report was investigating the potential overexpression of surface receptors, the current investigation sought to identify the relative expression of a given receptor across a wider panel of patient cell lines. This study further demonstrates that IR and PDGFR-*β* expressions appear to provide prognostic value, albeit within a limited context. IR was shown to be a significant predictor for EFS when stratified by metastatic status at diagnosis. Furthermore, amongst patients with nonmetastatic disease at initial diagnosis, high expression of IR significantly increased the hazard ratio for EFS when compared to intermediate IR expression. PDGFR-*β* was shown to be a significant predictor for EFS when stratified by metastatic status at diagnosis. Amongst patients whose disease was nonmetastatic at initial diagnosis, intermediate PDGFR-*β* expression significantly decreased the hazard ratio for EFS compared to low PDGFR-*β* expression while high PDGFR-*β* expression significantly increased the hazard ratio for EFS compared to intermediate PDGFR-*β* expression. Additionally, PDGFR-*β* was found to be a significant predictor of overall survival in patients with metastatic disease at diagnosis. Intermediate PDGFR-*β* expression significantly decreased the hazard ratio for overall survival compared to low PDGFR-*β* expression.

There are numerous reports that have characterized the role of the IR/IGF-1R signaling pathway in the tumorigenesis and metastasis of various cancers [12, 13]. Li et al. have implicated the pathway, demonstrating that overexpression of IGF-1R promotes cellular proliferation, cell survival, and drug resistance, subsequently leading to OS metastasis [14]. Wang et al. compared mRNA and protein expression levels of IGF-1R in 26 OS cell lines with noncancerous bone cell lines and found both mRNA and protein levels were significantly higher within the OS cell lines [15]. Additionally, they analyzed 84 OS cell lines to demonstrate the correlation between IGF-1R expression and survival. High IGF-1R expression was associated with poorer survival, with multivariate Cox analyses demonstrating it to be an independent prognostic marker. Only a handful of reports exist evaluating the IR signaling pathway in the context of OS, and none to our knowledge evaluating its use for prognosis in OS. The current study implicates IR as a prognostic marker for EFS in patients with OS; however, IGF-1R was not found to have a similar value.

Both IR and IGF-1R have been previously investigated as therapeutic targets. A number of *in vitro* studies have evaluated the effect of inhibition of the IR/IGF-1R signaling pathway. Zhi et al. demonstrated that *in vitro* cell growth was better inhibited by cotargeting IGF-1R and IR-A than by targeting IGF-1R alone [16]. Other authors have reported successful suppression of cell proliferation, migration, and invasion in OS cell lines using either miRNA, siRNA, or inhibitory antibodies that targeted IGF-1R, IR, and IR substrate 1 [13, 16–22]. Kolb et al. used R1507, an anti-IGF-1R antibody, in OS xenograft tumor models to delay tumor growth in 4 of 6 OS xenografts with significant improvement in EFS [23]. Anderson et al. conducted a multi-institutional phase 2 clinical study using robatumumab in patients with relapsed OS and Ewing sarcoma [24]. OS patients with resectable tumors realized a median overall survival of 20 months, while OS patients with unresectable tumors demonstrated a median overall survival of 8.2 months. The authors concluded that while IGF-1R was targetable, additional investigation into its utility was needed. Despite an early interest in IGF-1R in particular, inhibition of the IR/IGF-1R pathway has not yielded meaningful clinical results to date within the context of OS. Various explanations for this have been postulated including OS's redundant autocrine loops and the development of adaptive resistance among others. While it remains unclear to what extent the IR/IGF-1R pathway can be harnessed for the treatment of OS, IR appears to offer prognostic value.

Platelet-derived growth factor has been implicated in the tumorigenesis and metastasis of several solid tumors and shown to portend a poor prognosis [25, 26]. Its role in the progression and prognosis of OS has been investigated as well [27, 28]. Kubo et al. examined surgical specimens from 54 OS patients, comparing the level of PDGF (PDGF-AA, PDGF-*α*, PDGF-BB, and PDGF-*β*) receptor expression through immunohistochemistry to patient prognosis. They found PDGF-AA and PDGF-*α* receptors were correlated with inferior EFS (*p* < 0.05), while PDGF-BB and PDGF-*β* did not correlate to inferior EFS (*p*=0.15).

Imatinib has been utilized in both preclinical and early phase clinical studies. In the same study previously mentioned, Kubo et al. evaluated imatinib mesylate as a therapeutic agent for OS. However, excessively high doses were required to achieve cytotoxicity and pathway inhibition, making this therapeutic approach unfeasible. Yamaguchi et al. evaluated the *in vivo* antitumor effects of imatinib versus imatinib and doxorubicin in mice with heterotopically injected OS tumors. They demonstrated that combination therapy yielded synergistic effects, inhibiting cell proliferation [29]. The COG conducted a phase 2 clinical study looking at the effects of imatinib in children with refractory or relapsed solid tumors [30]. None of their OS patients demonstrated response according to Response Evaluation Criteria in Solid Tumors (RECIST) and, as such, were unsuccessful in showing imatinib to be an effective, single-agent treatment.

The current study did not demonstrate Her-2 to be predictive of EFS or overall survival when stratified by metastatic status at initial diagnosis. The prognostic value of Her-2 in OS has been debated in the past and remains controversial. Akatsuka et al. analyzed the immunohistochemical expression of Her-2 in 81 tumor cell lines from patients with nonmetastatic OS treated with surgery and chemotherapy [31]. They found that Her-2 overexpression was associated with both significantly increased EFS (72.2% vs. 45.6% at 5 years, *p*=0.03) and overall survival (79.7% vs. 58.2% at 5 years, *p*=0.03). Additionally, decreased levels of Her-2 increased the risk of adverse events and death (rate ratio: 2.24 and 2.54; 95% CI, 1.07–4.72 and 1.09–5.67, respectively). In contrast, Zhang et al. performed a meta-analysis evaluating the relationship between Her-2 expression and OS [32]. They identified 16 OS studies that provided survival outcomes and identified cell lines as being Her-2 positive or negative. Overexpression of Her-2 was associated with decreased overall survival in both biopsy and surgically removed specimens (HR = 2.07, 95% CI: 1.16–3.72, *p*=0.014; and HR = 2.02, 95% CI: 1.10–3.71, *p*=0.024). Finally, the COG conducted a large prospective study of 149 patients with newly diagnosed OS to determine the prognostic value of Her-2 [33]. They were unable to demonstrate that Her-2 status was associated with EFS or overall survival in patients with localized disease, concluding Her-2 expression was not prognostic, consistent with our findings.

Despite conflicting evidence regarding the prognostic utility of Her-2, its role as a therapeutic target has been pursued. *In vitro* studies by Long et al. investigated the role of lapatinib, an inhibitor of Her-2 phosphorylation, in standard OS cell lines [34]. They found a dose- and time-dependent inhibition of cellular proliferation, higher apoptotic rates, and inhibition of migratory/invasive abilities. Rainusso et al. utilized Her-2-specific CAR T cells to target tumor-initiating cells (TICs) in OS within an orthotopic xenograft model [35]. *In vivo* administration of the Her-2-specific T cells significantly reduced TICs, as evidenced by a reduction in sarcosphere forming efficiency in the explanted tumors. A phase 2 clinical trial, involving 96 patients with the newly diagnosed metastatic OS, sought to determine the safety and feasibility of trastuzumab as an adjunct to chemotherapy in patients whose tumors overexpressed Her-2 [36]. The 30-month EFS and OS for patients with Her-2 overexpression treated with chemotherapy and trastuzumab were 32% and 59%, respectively. Patients without Her-2 overexpression treated with chemotherapy alone demonstrated EFS and OS of 32% and 50%, respectively. These results failed to demonstrate significant improvement in survival by the addition of trastuzumab. While Her-2 remains a feasible target, further investigation into its clinical value is needed.

The MET signaling pathway has been well described and implicated in the epithelial-mesenchymal transition (EMT) of tumor cells [37]. In theory, expression of c-Met is expected to be predictive of worse outcome. However, the current study did not demonstrate the prognostic value of c-Met in predicting EFS or overall survival in patients with OS, and a review of the literature reveals a paucity of studies comparing c-Met expression and clinical outcomes in the context of OS.

c-Met has been investigated as a potential therapeutic target. *In vitro* studies utilizing miRNA to inhibit c-Met have been successful in preventing cell proliferation, migration, and invasion in OS [38, 39]. Cabozantinib, an inhibitor of c-Met, has been investigated in both preclinical and clinical settings. Fioramonti et al. showed that cabozantinib decreased OS cell proliferation and migration through its effects on OS cells and their microenvironment [40]. The French Sarcoma Group conducted a phase 2 combined clinical trial using cabozantinib in patients with advanced Ewing sarcoma or OS, to assess efficacy both histologically and radiographically [41]. Five of 42 patients (12%; 95% CI 4–26) with OS had objective responses by 6 months; 14 of 42 patients (33%; 95% CI 20–50) had 6-month nonprogression. They concluded that cabozantinib was well tolerated and demonstrated antitumor effects, warranting further investigation.

Vascular endothelial growth factor has been extensively reported on in the literature to be associated with poor prognosis in OS due to its promotion of angiogenesis and metastasis [42–45]. In contrast, our study was unsuccessful in demonstrating its use as a prognostic marker for EFS or overall survival in patients with OS.

Similar to the previously discussed receptors, VEGF has been investigated as a therapeutic target both *in vitro* and clinically. Studies have utilized a variety of miRNAs to inhibit the VEGF pathway and successfully suppressed cell proliferation, invasion, and angiogenesis in standard OS cell lines [46–48]. Grignani et al. conducted a nonrandomized, phase 2 clinical trial assessing the efficacy of sorafenib, an anti-VEGF antibody, and everolimus in patients with unresectable high-grade OS that had progressed despite standard chemotherapy treatment [49]. Of the 38 patients enrolled, 17 were progression-free at six months (45%; 95% CI 28–61). They failed to demonstrate that treatment with sorafenib and everolimus improved disease progression at six months, despite having a small proportion of patients who were progression-free. Navid et al. completed phase 2 clinical trials to evaluate the role of bevacizumab as an adjunct to standard OS treatment [50]. Thirty-one patients with localized OS received bevacizumab and chemotherapy both pre- and postoperatively. The estimated 4-year EFS and overall survival rate were 57.5 ± 10% and 83.4 ± 7.8 %, respectively. They concluded that while bevacizumab is a tolerable adjunctive therapy, the histologic tumor responses and EFS did not support further investigation.

This study is limited by several factors. Most importantly, the experimental environment does not adequately recapitulate the human experience; *in vitro* tumors do not entirely reflect the *in vivo* state. Moreover, receptor expression was tested at a single point in time, assuming that expression is stable over time both *in vitro* and *in vivo*. Lastly, we analyzed a small sample from a larger tumor that is known to be genomically heterogeneous. Findings may be limited by sampling error, yielding results that may not be representative of the whole tumor.

In summary, this study demonstrated variable expression of all six surface receptors across all patient-derived OS cell lines analyzed. Furthermore, IR and PDGFR-*β* expression levels demonstrated prognostic value in predicting EFS and overall survival. To our knowledge, this study is the first to characterize expression levels and prognostic value of multiple cell surface receptors across a large panel of patient-derived OS cell lines. It remains unclear whether one or more of these receptors can be leveraged in a targeted therapeutic manner; however, their consistent, albeit variable expression may permit for such an opportunity. Since osteosarcoma's genomic variability and high mutational burden makes it unlikely that a single treatment will adequately address all relapsed, metastatic, and/or chemoresistant cases. One, or a combination, of the receptors discussed may indeed prove useful in future targeted approaches and further investigation into such strategies is warranted.

## Figures and Tables

**Table 1:** Summary of antibodies and their respective isotype control antibodies.

Receptor antibody (fluorophore)	Vendor/catalog no.	Isotype antibody	Positive control
Monoclonal antihuman HER-2/neu (PE)	BD/340552	Mouse IgG1/Neu24.7	MCF7 (ATCC/HTB-22)
Monoclonal antihuman CD140b/PDGFR-*β* (PE)	R&D/FAB1263P	Mouse IgG1/PR7212	Hs 697.Sp (ATCC/CRL-7433)
Monoclonal antihuman IGF-1R (FITC)	R&D/FAB391F	Mouse IgG1/33255	MCF7 ATCC/HTB-22)
Polyclonal antihuman IR (AF488)	R&D/FAB1544G	Goat IgG/NP_001073285	Monocytes (periph blood sample)
Monoclonal antihuman HGFR/c-MET (APC)	R&D/FAB3582A	Mouse IgG1/95106	Monocytes (periph blood sample)
Monoclonal antihuman VEGFR-3 (APC)	R&D/FAB3492A	Mouse IgG1/54733	Monocytes (periph blood sample)

**Table 2:** Summary of patient demographic data.

Demographics	Total	
Metastasis		
Nonmetastatic	31	64.6%
Metastatic	17	35.4%

Sex		
Male	31	64.6%
Female	17	35.4%

Race		
Black	10	20.8%
Unknown	3	6.3%
White	35	72.9%

Ethnicity		
Hispanic	7	14.6%
Non-Hispanic	40	83.3%
Unknown	1	2.1%

Age category		
1 to 9 years old	9	18.8%
≥10 years old	39	81.3%

## Data Availability

Data sharing is not applicable to this article as no new data were created or analyzed in this study.

## Supplementary Materials

Supplementary Materials Supplementary Table 1: number of EFS events per patient with respect to low, intermediate, or high receptor expression. Supplementary Table 2: number of OS events per patient with respect to low, intermediate, or high receptor expression. Supplementary Table 3: raw flow cytometry expression data for six surface receptors across all cell lines, expressed in geometric mean fluorescent intensity difference (geoMFI_diff_). Supplementary Table 4: expression data for six surface receptors across all cell lines, expressed in low, intermediate, or high. Supplementary Figure 1: receptor expression patterns for Her-2 (A), PDGFR-*β* (B), IGF-1R (C), IR (D), cMet (E), and VEGFR-3 (F) across all cell lines expressed in geoMFI_diff_. Supplementary Table 5: EFS hazard ratios for IR expression both when analyzing all patients included in the study and a subgroup analysis of metastatic and nonmetastatic patients. Supplementary Table 6: overall survival hazard ratios for IR expression both when analyzing all patients included in the study and a subgroup analysis of metastatic and nonmetastatic patients. Supplementary Table 7: EFS hazard ratios for PDGFR-*β* expression both when analyzing all patients included in the study and a subgroup analysis of metastatic and nonmetastatic patients. Supplementary Table 8: overall survival hazard ratios for PDGFR-*β* expression both when analyzing all patients included in the study and a subgroup analysis of metastatic and nonmetastatic patients.
