# Supplementary material for: Prognostic and Therapeutic Utility of Variably Expressed Cell Surface Receptors in Osteosarcoma
Source: Sarcoma. 2021 Feb 2;2021:8324348. doi: 10.1155/2021/8324348 (PMC7872755; doi:10.1155/2021/8324348)
Supplement: Supplementary Materials — Supplementary Table 1: number of EFS events per patient with respect to low, intermediate, or high receptor expression. Supplementary Table 2: number of OS events per patient with respect to low, intermediate, or high receptor expression. Supplementary Table 3: raw flow cytometry expression data for six surface receptors across all cell lines, expressed in geometric mean fluorescent intensity difference (geoMFIdiff). Supplementary Table 4: expression data for six surface receptors across all cell lines, expressed in low, intermediate, or high. Supplementary Figure 1: receptor expression patterns for Her-2 (A), PDGFR-β (B), IGF-1R (C), IR (D), cMet (E), and VEGFR-3 (F) across all cell lines expressed in geoMFIdiff. Supplementary Table 5: EFS hazard ratios for IR expression both when analyzing all patients included in the study and a subgroup analysis of metastatic and nonmetastatic patients. Supplementary Table 6: overall survival hazard ratios for IR expression both when analyzing all patients included in the study and a subgroup analysis of metastatic and nonmetastatic patients. Supplementary Table 7: EFS hazard ratios for PDGFR-β expression both when analyzing all patients included in the study and a subgroup analysis of metastatic and nonmetastatic patients. Supplementary Table 8: overall survival hazard ratios for PDGFR-β expression both when analyzing all patients included in the study and a subgroup analysis of metastatic and nonmetastatic patients. [file 8324348.f1.docx]

**Supplementary Table 1.** Number of EFS events per patient with respect to low, intermediate or high receptor expression

| **Analysis Variables** | **EFS Event** | | **Total** |
| --- | --- | --- | --- |
|  | **No** | **Yes** |  |
| **Her2** |  |  |  |
| **Low** | 1 | 10 | 11 |
| **Intermediate** | 11 | 14 | 25 |
| **High** | 5 | 7 | 12 |
| **PDGFRB** |  |  |  |
| **Low** | 1 | 10 | 11 |
| **Intermediate** | 12 | 14 | 26 |
| **High** | 4 | 7 | 11 |
| **IGFIR** |  |  |  |
| **Low** | 5 | 8 | 13 |
| **Intermediate** | 8 | 17 | 25 |
| **High** | 4 | 6 | 10 |
| **IR** |  |  |  |
| **Low** | 5 | 7 | 12 |
| **Intermediate** | 11 | 14 | 25 |
| **High** | 1 | 10 | 11 |
| **cMet** |  |  |  |
| **Low** | 4 | 9 | 13 |
| **Intermediate** | 10 | 13 | 23 |
| **High** | 3 | 9 | 12 |
| **VEGFR3** |  |  |  |
| **Low** | 5 | 7 | 12 |
| **Intermediate** | 9 | 15 | 24 |
| **High** | 3 | 9 | 12 |

**Supplementary Table 2.** Number of OS events per patient with respect to low, intermediate or high receptor expression.

| **Analysis Variables** | **OS Event** | | **Total** |
| --- | --- | --- | --- |
|  | **Alive** | **Dead** |  |
| **Her2** |  |  |  |
| **Low** | 5 | 6 | 11 |
| **Intermediate** | 16 | 9 | 25 |
| **High** | 9 | 3 | 12 |
| **PDGFRB** |  |  |  |
| **Low** | 3 | 8 | 11 |
| **Intermediate** | 18 | 8 | 26 |
| **High** | 9 | 2 | 11 |
| **IGFIR** |  |  |  |
| **Low** | 8 | 5 | 13 |
| **Intermediate** | 16 | 9 | 25 |
| **High** | 6 | 4 | 10 |
| **IR** |  |  |  |
| **Low** | 8 | 4 | 12 |
| **Intermediate** | 16 | 9 | 25 |
| **High** | 6 | 5 | 11 |
| **cMet** |  |  |  |
| **Low** | 9 | 4 | 13 |
| **Intermediate** | 15 | 8 | 23 |
| **High** | 6 | 6 | 12 |
| **VEGFR3** |  |  |  |
| **Low** | 8 | 4 | 12 |
| **Intermediate** | 16 | 8 | 24 |
| **High** | 6 | 6 | 12 |

**Supplementary Table 3.** Raw flow cytometry expression data for six surface receptors across all cell lines, expressed in geometric mean fluorescent intensity difference (geoMFI_diff_)

|  | **Her2** | **PDGFRB** | **IGFIR** | **IR** | **cMet** | **VEGFR3** |
| --- | --- | --- | --- | --- | --- | --- |
| C194 | 3539 | 1998 | 94 | 145 | 917 | 27 |
| C204 | 2249 | 465 | 64 | 81 | 1456 | -24 |
| C205 | 9417 | 3002 | 107 | 186 | 4027 | 78 |
| C209 | 3738 | 3443 | 45 | 181 | 2678 | 37 |
| C219 | 3544 | 4365 | 113 | 94 | 982 | 44 |
| C231 | 3559 | 2021 | 9 | 138 | 420 | 7 |
| C232 | 2688 | 2791 | 153 | 168 | 343 | 28 |
| C233 | 3840 | 2627 | 177 | 165 | 903 | 44 |
| C238 | 3090 | 3352 | 133 | 181 | 459 | 37 |
| C243 | 1500 | 2139 | 78 | 166 | 292 | -12 |
| C244 | 2210 | 5440 | 79 | 209 | 240 | 61 |
| C251 | 5726 | 8915 | 115 | 233 | 981 | 35 |
| C253 | 3953 | 6728 | 87 | 327 | 2343 | 69 |
| C256 | 2069 | 665 | 64 | 106 | 2579 | 6 |
| C281 | 2920 | 1691 | 138 | 157 | 1296 | 91 |
| C282 | 5893 | 12265 | 282 | 146 | 147 | 81 |
| C287 | 1716 | 5435 | 152 | 216 | 665 | 122 |
| C290 | 3157 | 4165 | 127 | 89 | 363 | 12 |
| C291 | 3552 | 4422 | 43 | 216 | 3296 | 65 |
| C293 | 2785 | 1529 | 40 | 91 | 408 | 9 |
| C297 | 3626 | 1007 | 29 | 86 | 1959 | 3 |
| C299 | 4094 | 1772 | 121 | 97 | 834 | 36 |
| C300 | 2788 | 392 | 35 | 93 | 2688 | 57 |
| C301 | 1724 | 934 | 62 | 89 | 128 | 31 |
| C307 | 3741 | 919 | 238 | 173 | 2362 | 86 |
| C311 | 2377 | 4256 | 91 | 181 | 446 | 21 |
| C314 | 1285 | 1703 | 46 | 83 | 535 | 6 |
| C315 | 2922 | 3570 | 131 | 133 | 975 | 26 |
| C323 | 7838 | 4510 | 274 | 129 | 1596 | 74 |
| C326 | 3969 | 3906 | 139 | 111 | 498 | 52 |
| C327 | 6873 | 12434 | 60 | 178 | 223 | 27 |
| C331 | 1808 | 932 | 43 | 96 | 698 | 25 |
| C334 | 2869 | 2811 | 57 | 101 | 469 | 34 |
| C337 | 2534 | 862 | 91 | 100 | 1314 | 36 |
| C338 | 4532 | 4442 | 65 | 173 | 219 | 6 |
| C340 | 3813 | 11289 | 57 | 92 | 418 | -6 |
| C342 | 2907 | 4684 | 354 | 407 | 249 | 69 |
| C346 | 1800 | 829 | 19 | 86 | 777 | 26 |
| C349 | 4634 | 5893 | 131 | 208 | 1034 | 54 |
| C353 | 3035 | 1461 | 85 | 245 | 400 | 65 |
| C360 | 2525 | 800 | 92 | 153 | 816 | 18 |
| C365 | 6032 | 8191 | 32 | 93 | 1163 | -2 |
| C366 | 4124 | 9145 | 26 | 92 | 390 | 27 |
| C368 | 4908 | 6079 | 93 | 137 | 322 | 17 |
| C370 | 9968 | 22800 | 65 | 356 | 5914 | 3 |
| C371 | 6633 | 10511 | 75 | 259 | 722 | 7 |
| C373 | 10058 | 44048 | 171 | 478 | 826 | 21 |
| C374 | 12287 | 25500 | 142 | 375 | 1599 | 62 |
| C375 | 1821 | 4678 | 47 | 103 | 215 | 39 |
| C377 | 2902 | 3749 | 82 | 143 | 427 | 21 |
| C379 | 3656 | 4977 | 87 | 120 | 663 | 37 |
| C396 | 16671 | 21343 | 220 | 402 | 7105 | 16 |

**Supplementary Table 4.** Expression data for six surface receptors across all cell lines, expressed in low, intermediate, or high.

|  | **Her2** | **PDGFRB** | **IGFIR** | **IR** | **cMet** | **VEGFR3** |
| --- | --- | --- | --- | --- | --- | --- |
| C194 | Intermediate | Intermediate | Intermediate | Intermediate | Intermediate | Intermediate |
| C204 | Low | Low | Intermediate | Low | High | Low |
| C205 | High | Intermediate | Intermediate | Intermediate | High | High |
| C209 | Intermediate | Intermediate | Low | Intermediate | High | Intermediate |
| C219 | Intermediate | Intermediate | Intermediate | Low | Intermediate | Intermediate |
| C231 | Intermediate | Intermediate | Low | Intermediate | Intermediate | Low |
| C232 | Intermediate | Intermediate | Intermediate | Intermediate | Low | Intermediate |
| C233 | Intermediate | Intermediate | Intermediate | Intermediate | Intermediate | Intermediate |
| C238 | Intermediate | Intermediate | Intermediate | Intermediate | Intermediate | Intermediate |
| C243 | Low | Intermediate | Intermediate | Intermediate | Low | Low |
| C244 | Low | Intermediate | Intermediate | High | Low | High |
| C251 | High | High | Intermediate | High | Intermediate | Intermediate |
| C253 | Intermediate | High | Intermediate | High | High | High |
| C256 | Low | Low | Intermediate | Intermediate | High | Low |
| C281 | Intermediate | Low | High | Intermediate | Intermediate | High |
| C282 | High | High | High | Intermediate | Low | High |
| C287 | Low | Intermediate | High | High | Intermediate | High |
| C290 | Intermediate | Intermediate | Intermediate | Low | Low | Low |
| C291 | Intermediate | Intermediate | Low | High | High | High |
| C293 | Intermediate | Low | Low | Low | Intermediate | Low |
| C297 | Intermediate | Low | Low | Low | High | Low |
| C299 | Intermediate | Intermediate | Intermediate | Intermediate | Intermediate | Intermediate |
| C300 | Intermediate | Low | Low | Low | High | High |
| C301 | Low | Low | Intermediate | Low | Low | Intermediate |
| C307 | Intermediate | Low | High | Intermediate | High | High |
| C311 | Low | Intermediate | Intermediate | Intermediate | Intermediate | Intermediate |
| C314 | Low | Intermediate | Low | Low | Intermediate | Low |
| C315 | Intermediate | Intermediate | Intermediate | Intermediate | Intermediate | Intermediate |
| C323 | High | Intermediate | High | Intermediate | High | High |
| C326 | Intermediate | Intermediate | High | Intermediate | Intermediate | Intermediate |
| C327 | High | High | Intermediate | Intermediate | Low | Intermediate |
| C331 | Low | Low | Low | Low | Intermediate | Intermediate |
| C334 | Intermediate | Intermediate | Low | Intermediate | Intermediate | Intermediate |
| C337 | Low | Low | Intermediate | Intermediate | Intermediate | Intermediate |
| C338 | Intermediate | Intermediate | Intermediate | Intermediate | Low | Low |
| C340 | Intermediate | High | Low | Low | Intermediate | Low |
| C342 | Intermediate | Intermediate | High | High | Low | High |
| C346 | Low | Low | Low | Low | Intermediate | Intermediate |
| C349 | High | Intermediate | Intermediate | High | Intermediate | Intermediate |
| C353 | Intermediate | Low | Intermediate | High | Low | High |
| C360 | Low | Low | Intermediate | Intermediate | Intermediate | Intermediate |
| C365 | High | High | Low | Low | Intermediate | Low |
| C366 | Intermediate | High | Low | Low | Low | Intermediate |
| C368 | High | High | Intermediate | Intermediate | Low | Intermediate |
| C370 | High | High | Intermediate | High | High | Low |
| C371 | High | High | Intermediate | High | Intermediate | Low |
| C373 | High | High | High | High | Intermediate | Intermediate |
| C374 | High | High | High | High | High | High |
| C375 | Low | Intermediate | Low | Intermediate | Low | Intermediate |
| C377 | Intermediate | Intermediate | Intermediate | Intermediate | Intermediate | Intermediate |
| C379 | Intermediate | Intermediate | Intermediate | Intermediate | Intermediate | Intermediate |
| C396 | High | High | High | High | High | Intermediate |

**Supplementary Figure 1.** Receptor expression patterns for Her2 (A), PDGFRβ (B), IGF-1R (C), IR (D), cMet (E), VEGFR-3 (F) across all cell lines expressed in GeoMFI_diff_

__

**Supplementary Table 5.** EFS hazard ratios for IR expression both when analyzing all patients included in the study, as well as a sub-group analysis of metastatic and non-metastatic patients.

| **Analysis Variable**  **(Value Ratio)** | **Hazard Ratio** | **95% Confidence Interval for HR** | ***p*-value for HR ≠ 1** |
| --- | --- | --- | --- |
| **All Patients*** |  |  |  |
| **(Intermediate vs. Low)** | 0.896 | (0.358, 2.244) | 0.8150 |
| **(High vs. Low)** | 2.381 | (0.900, 6.298) | 0.0806 |
| **(High vs. Intermediate)** | 2.656 | (1.140, 6.189) | 0.0236 |
| **Non-Metastatic Only** |  |  |  |
| **(Intermediate vs. Low)** | 0.695 | (0.202, 2.389) | 0.5635 |
| **(High vs. Low)** | 3.112 | (0.867, 11.165) | 0.0816 |
| **(High vs. Intermediate)** | 4.477 | (1.426, 14.057) | 0.0102 |
| **Metastatic Only** |  |  |  |
| **(Intermediate vs. Low)** | 1.231 | (0.305, 4.963) | 0.7703 |
| **(High vs. Low)** | 1.777 | (0.354, 8.924) | 0.4849 |
| **(High vs. Intermediate)** | 1.444 | (0.358, 5.824) | 0.6057 |

*Metastatic at Diagnosis was also a predictor variable in the model.

**Supplementary Table 6.** Overall survival hazard ratios for IR expression both when analyzing all patients included in the study, as well as a sub-group analysis of metastatic and non-metastatic patients.

| **Analysis Variable**  **(Value Ratio)** | **Hazard Ratio** | **95% Confidence Interval for HR** | ***p*-value for HR ≠ 1** |
| --- | --- | --- | --- |
| **All Patients*** |  |  |  |
| **(Intermediate vs. Low)** | 1.280 | (0.392, 4.176) | 0.6829 |
| **(High vs. Low)** | 2.467 | (0.656, 9.284) | 0.1817 |
| **(High vs. Intermediate)** | 1.928 | (0.641, 5.797) | 0.2425 |
| **Non-Metastatic Only** |  |  |  |
| **(Intermediate vs. Low)** | 1.930 | (0.215, 17.288) | 0.5568 |
| **(High vs. Low)** | 3.271 | (0.295, 36.232) | 0.3341 |
| **(High vs. Intermediate)** | 1.695 | (0.307, 9.355) | 0.5448 |
| **Metastatic Only** |  |  |  |
| **(Intermediate vs. Low)** | 2.324 | (0.456, 11.842) | 0.3099 |
| **(High vs. Low)** | 1.045 | (0.248, 4.414) | 0.9517 |
| **(High vs. Intermediate)** | 2.223 | (0.514, 9.625) | 0.2852 |

*Metastatic at Diagnosis was also a predictor variable in the model.

**Supplementary Table 7.** EFS hazard ratios for PDGFRβ expression both when analyzing all patients included in the study, as well as a sub-group analysis of metastatic and non-metastatic patients.

| **Analysis Variable (Value Ratio)** | **Hazard Ratio** | **95% Confidence Interval for HR** | ***p*-value for HR ≠ 1** |
| --- | --- | --- | --- |
| **All Patients*** |  |  |  |
| **(Intermediate vs. Low)** | 0.377 | (0.163, 0.873) | 0.0228 |
| **(High vs. Low)** | 2.141 | (0.664, 6.901) | 0.2024 |
| **(High vs. Intermediate)** | 5.678 | (1.833, 17.584) | 0.0026 |
| **Non-Metastatic Only** |  |  |  |
| **(Intermediate vs. Low)** | 0.414 | (0.120, 1.431) | 0.1632 |
| **(High vs. Low)** | 2.588 | (0.606, 11.041) | 0.1990 |
| **(High vs. Intermediate)** | 6.254 | (1.725, 22.667) | 0.0053 |
| **Metastatic Only**** |  |  |  |
| **(Intermediate vs. Low)** | 0.419 | (0.133, 1.323) | 0.1382 |

*Metastatic at Diagnosis was also a predictor variable in the model.

**There were no Metastatic at Diagnosis patients with High level PDGFRB

**Supplementary Table 8.** Overall survival hazard ratios for PDGFRβ expression both when analyzing all patients included in the study, as well as a sub-group analysis of metastatic and non-metastatic patients.

| **Analysis Variable (Value Ratio)** | **Hazard Ratio** | **95% Confidence Interval for HR** | ***p*-value for HR ≠ 1** |
| --- | --- | --- | --- |
| **All Patients*** |  |  |  |
| **(Intermediate vs. Low)** | 0.310 | (0.112, 0.856) | 0.0237 |
| **(High vs. Low)** | 1.014 | ( 0.172, 5.966) | 0.9880 |
| **(High vs. Intermediate)** | 3.270 | (0.535, 19.978) | 0.1995 |
| **Non-Metastatic Only** |  |  |  |
| **(Intermediate vs. Low)** | 0.452 | (0.075, 2.722) | 0.3858 |
| **(High vs. Low)** | 1.056 | (0.133, 8.387) | 0.9586 |
| **(High vs. Intermediate)** | 2.339 | (0.360, 15.197) | 0.3734 |
| **Metastatic Only**** |  |  |  |
| **(Intermediate vs. Low)** | 0.261 | (0.065, 1.052) | 0.0590 |

*Metastatic at Diagnosis was also a predictor variable in the model.

**There were no Metastatic at Diagnosis patients with High level PDGFRB
